# A Survey on One Health Perception and Experiences in Europe and Neighboring Areas

**DOI:** 10.3389/fpubh.2021.609949

**Published:** 2021-02-17

**Authors:** Francesco Chiesa, Laura Tomassone, Sara Savic, Alessandro Bellato, Andrei Daniel Mihalca, David Modry, Barbara Häsler, Daniele De Meneghi

**Affiliations:** ^1^Department of Veterinary Science, University of Turin, Grugliasco-Turin, Italy; ^2^Scientific Veterinary Institute “Novi Sad”, Novi Sad, Serbia; ^3^Faculty of Veterinary Medicine, University of Agricultural Sciences and Veterinary Medicine of Cluj-Napoca, Cluj Napoca, Romania; ^4^Department of Botany and Zoology, Faculty of Science, Masaryk University, Brno, Czechia; ^5^Institute of Parasitology, Biology Centre, Czech Academy of Sciences, Ceske Budejovice, Czechia; ^6^Department of Veterinary Sciences, Faculty of Agrobiology, Food and Natural Resources/CINeZ, Czech University of Life Sciences Prague, Prague, Czechia; ^7^Royal Veterinary College, London, United Kingdom

**Keywords:** questionnaire survey, One Health, EU COST countries, intersectoral collaboration, interdisciplinary/multidisciplinary, OH strategies and policies

## Abstract

A questionnaire survey was promoted under the COST Actions “*Network for Evaluation of One Health-NEOH”* and “*European Network for Neglected Vectors and Vector-Borne Infections-EURNEGVEC”*, from June 2016 to April 2017, to collect information on the existence of One Health (OH) collaboration and implementation of OH initiatives in 37 EU COST Countries. The questionnaire was to be answered by key respondents representing the three major OH components: (i). Animal Health; (ii). Human Health/Public Health; (iii). Environmental Health. A target respondent rate of nine respondents/country was aimed for, representing the following categories: (i). ministries; (ii). academia-research; (iii). private sector and NGOs, associations and scientific societies. The questionnaire, composed of 27 questions organized in six sections, was circulated to target respondents by Committee Members of the two COST actions. A total of 171 respondents from 34 countries completed the questionnaire, mainly belonging to academic and research institutions (55.5%), and to Animal Health/Animal Science fields (53.8%). Although the majority (57.9%) declared they had heard about OH, few respondents (10.7%) provided a complete definition. The “human” and “animal” elements prevailed over other key elements of OH definition (ecosystem, intersectoral, transdisciplinary, holistic, collaboration). Overall, 62.6% respondents declared to take part in OH initiatives. Antimicrobial resistance, avian influenza and environmental pollution were cited as the top three OH issues over the past 5 years. Limitations and gaps in intersectoral collaboration included communication and organizational problems resulting in poor networking, differing priorities and a lack of understanding between sectors. Regarding control and monitoring of zoonotic diseases, respondents from different sectors preferentially selected their own directorates/ministries while actually in most countries both Ministry of Health and Ministry of Agriculture are engaged. According to respondents, the level of awareness of OH amongst the general public is limited. Similarly, a dearth of opportunities of collaborations at different institutional and/or professional levels was described. Our survey provided an overview of how respondents in COST countries perceived and experienced OH and current limits to OH implementation. Identifying how initiatives are currently working and knowing the promoting and hindering factors allowed suggesting strategies to promote efficiency and effectiveness of OH implementation in the future.

## Introduction

The collaboration between human, animal and environmental health sectors is considered crucial; a need highlighted by recent financial, economic, social, environmental and health crises. The complexity of health determinants, makes it difficult for single disciplines and institutions to deal with all health problems and challenges related to animal, public and environmental health (1, 2).

Changes in land use, habitat fragmentation, ecosystem invasion, direct and indirect interactions between animal species, including ourselves, have increased, and this has led to changes to ecological or biological systems. This fact has greatly contributed to the emergence of zoonotic pathogens: changes in patterns of contact between wild and domestic animals (e.g., Nipah virus), direct human and wild animal contact (e.g., HIV, Ebola), and changes in species abundance or diversity (e.g., Hantavirus, Lyme disease) (3). New pathogens might have the capacity for inter-human spread and even pandemic potential among humans, as observed in the current COVID-19 global emergency. Outbreaks of highly infectious diseases, including zoonoses, but also antibiotic resistance, interrelated obesity in humans and pets, food security and food safety, problems related to growing urbanization, lack of green spaces in the cities, uncontrolled environmental pollution and biodiversity loss, are only few examples where an integrated approach to health, such as One Health (OH), can be effectively employed (1, 2). Not only “classical zoonoses” can be tackled using a OH approach, but also other hazards occurring at the human, animal, ecosystem interface, such as exposure to toxicants through the environment and foods of animal origin. Some authors even advocate this new perspective as “toxicant-related zoonoses” highlighting the importance of new food safety issues within the environment-feed-food chain (4). Also, climate change adds uncertainty and contributes to health crises, affecting biological systems through various mechanisms, such as modifying the lifecycle of vectors, host species and pathogens, disrupting the synchrony among species, destructing habitats, etc. (5). Since species experience environmental changes differently, the expertise from veterinary, environmental and public health professionals are needed to understand ecological interactions and to forecast plausible reactions. The integration of expertise from environmental and life sciences can help to deepen knowledge and to understand each aspect of an issue, combining different perspectives (6).

In Europe, but also elsewhere, the degree and quality of collaboration amongst various health disciplines and institutions varies substantially. Integrated approaches to health are challenging because they require complex systems of communication and collaboration that are difficult to delimit (1). Despite the above-mentioned difficulties, in many European countries, a large number of integrated health initiatives has been implemented, some of them described or reported as “One Health.” Several studies investigated OH characteristics, provided proof of concept, demonstrated its added value, or established how to evaluate OH initiatives (7–14). Moreover, the World Bank published guidance on how to operationalize OH (15). Similarly, national action plans to combat AMR are encouraged to use OH principles (16). These efforts have generated momentum in OH and led to many different initiatives worldwide. A study looking at OH networks globally found a multitude of different networks, but generally a lack of direction and institutionalization (17). There is currently no register in Europe that would allow gaining a systematic understanding of the OH landscape in Europe.

In order to explore existing collaboration amongst the animal health, human/public health, environmental health and sectors, and to collect information on the existence and implementation of OH actions and initiatives in EU countries, a questionnaire-based survey was designed and circulated amongst EU COST Member Countries, associated and near-neighbor countries (https://www.cost.eu); there were 37 countries at the time the survey was initiated. The questionnaire was designed and promoted under the initiatives of the COST Actions TD1404 “Network for Evaluation of One Health-NEOH” (http://neoh.onehealthglobal.net) and TD1303 “European Network for Neglected Vectors and Vector-Borne Infections-EURNEGVEC” (https://www.eurnegvec.org).

## Materials and Methods

The questionnaire was developed in Google forms and structured in six sections: (1). *general information*; (2). *about “One Health”*; (3). *zoonotic diseases, environmental health and AMR: examples of “burning” OH issues/initiatives*; (4). *aspects limiting interdisciplinarity and intersectorality in OH*; (5). *conclusions*; (6). *end of questionnaire* (including comments, remarks and/or suggestions).

The survey consisted of 27 questions addressing the above mentioned objectives (i.e., to explore the existing collaboration amongst animal health, human/public health, environmental health sectors, to collect information on the existence and implementation of OH actions and initiatives in EU COST countries). Twenty-one were closed-ended questions, of which six in Likert-scale format, and six were open-ended questions. An informed consent form was provided at the beginning of the questionnaire where respondents were advised that the questionnaire was anonymous and that, by completing and submitting it, they voluntarily agreed to participate. Assurance of privacy and confidentiality is highly valued in e-mail questionnaire surveys, as reported by Saleh and Bista (18). Ethical approval was sought and granted by the Clinical Research and Ethical Review Board at the Royal Veterinary College, grant holder of COST Action TD1404 NEOH (ref. n. URN 2016 1554). Respondents were to be contacted by the Management Committee (MC) members and MC substitutes of the two COST Actions in each country. Key respondents were meant to represent the three components of OH (animal health, human health/public health and environmental health). MC members and MC substitutes contacted by email the target institutions through the official institutional contact email address, providing the link to the online questionnaire, and explaining to potential respondents the scope and the importance of the survey. Where deemed necessary, the COST Action members translated the message to their local language before reaching out to their contacts in the different institutions.

We expected nine respondents per country, one for each OH component, per each one of the following categories:

- Public institutions/ministries, that is, the Directors/Heads of Veterinary Services, Ministry of Agriculture (MoA), Ministry of Health (MoH); Directors/Heads of Human Health & Public Health Services, MoH; Environmental Health Services, Ministry of Environment or other applicable Ministries/Directorates, according to the national organization/system of each COST countries;
- Academia/research (i.e., Deans/Directors of the Schools/Universities and of National Research Centers on Veterinary Medicine, Human Medicine and Public Health, and Environmental/Earth Sciences, under Ministry of Education;
- Private sector (i.e., presidents of the National Boards/Colleges of Veterinarians, Medical Surgeons & Public Health doctors, and Environmental Earth Sciences doctors.

In addition, NGOs, associations, scientific societies involved in OH initiatives and activities were asked to participate in the survey.

The overall expected respondents were 333 (three respondents representing human, animal and environmental health each from at least three of the four categories listed per 37 COST countries).

The questionnaire was accessible for 10 months (June 2016–April 2017); it was subsequently closed, and the data collected were downloaded. Answers were checked for consistency, cleaned for analysis and the questionnaire was removed from the hosting platform.

A blank version of the questionnaire (.pdf) is available in the Supplementary Material.

Data were analyzed with R software (19). We computed descriptive statistics of questions answers and scores. In order to analyze qualitative data, open answers were categorized into classes. Non-parametric tests (Wilcoxon test and Kruskal-Wallis rank sum test) were used to detect significant differences in scores among categorical variables; Chi-square and Fisher Exact tests were employed to evaluate associations among categorical variables. For all statistical tests, a two-tailed significance level of α = 0.05 was adopted.

As regards the “definition of One Health” (question in section 2), answers were analyzed in order to detect five key words categories in the definition given by respondents, and a score was attributed, being five the maximum (all key words and key word categories included in the definition). The five key words were human health, environmental health, animal health, intersectoral/transdisciplinary/holistic, collaboration/sharing, as described in literature (2, 20). Advantages of OH, which respondents were asked to score (question in section 2), are described in Häsler et al. (21).

Interviewees were asked to select health hazards (mainly zoonotic diseases) that are controlled and monitored by the Ministry of Health and/or Agriculture (question in section 3); the list of major health hazards/zoonoses was taken from the Public Health England website (https://www.gov.uk/government/publications/list-of-zoonotic-diseases/list-of-zoonotic-diseases).

In section 4, interviewees were asked to score the level and opportunities for OH collaborations in their countries at different institutional and/or professional levels, choosing among: “poor,” “fair,” “good,” “excellent,” and “n/a”: not applicable.

## Results

Overall, 171 key respondents from 34 countries answered the questionnaire with at least one respondent per country.

**Figure 1:**
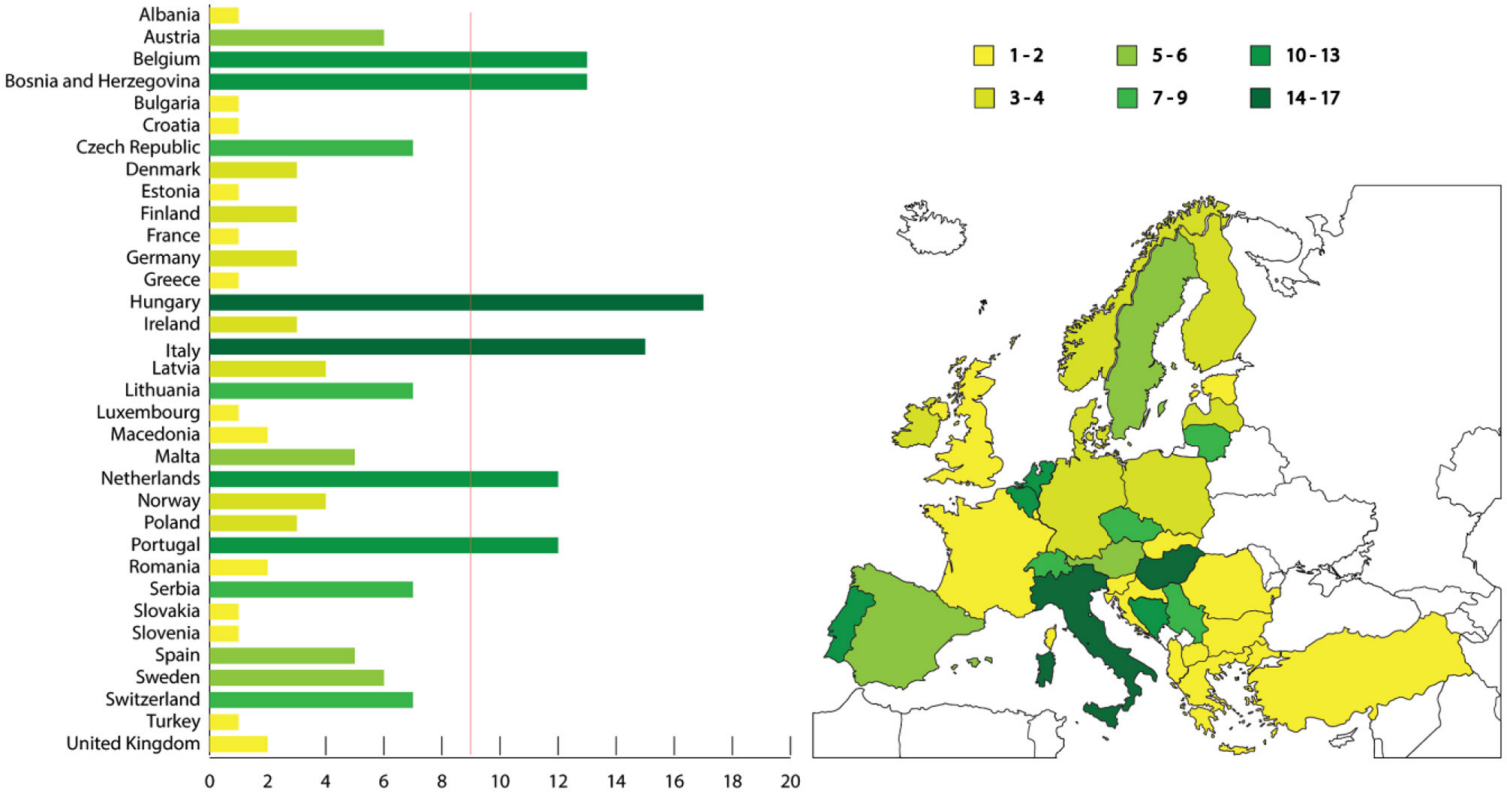
Map illustrating the number of respondents to the questionnaire per country, and bar chart with the number of questionnaires answered considering the number of expected respondents (red line). Colors grade from yellow (low number of questionnaires answered) to dark green (high number).

Few countries -namely Belgium, Bosnia & Herzegovina, Hungary, Italy, The Netherlands, and Portugal- reached or even exceeded, the expected minimum number of questionnaires answered (*n* = 9/country), other countries reached or slightly exceeded six questionnaires answered (Austria, Czech Republic, Lithuania, Serbia and Switzerland), while the remaining others answered less. No responses were received from Iceland, Montenegro, and Cyprus. The number of respondents by country is shown in Figure 1.

Results are reported by questionnaire section.

### Questionnaire Section 1—General Information

The majority of respondents declared to have training or a professional background in Animal Health or Animal Sciences (*n* = 92; 53.8%), followed by Public Health or Human Health (*n* = 51; 29.8%). Those having a professional background in Environmental Sciences and Life Sciences (including biologists, chemists and basic science disciplines) were respectively 12 (9.3%) and 5 (2.9%). Five respondents (2.9%) had a professional background in Food Safety, while two respondents were trained outside the “traditional” health disciplines, one of them in Sociology (0.6%) and the other one in Engineering (0.6%).

Most respondents worked at Higher Education Institutions/Universities (*n* = 66; 38.6%) and Research Centers (*n* = 29; 17.0%), followed by Governmental Institutions/Ministries (*n* = 44; 25.7%). Those working in the private sector and NGOs were 14 (8.2%) and 9 (5.3%), respectively. Two respondents were employed in International Organizations, and seven respondents did not give details.

**Figure 2:**
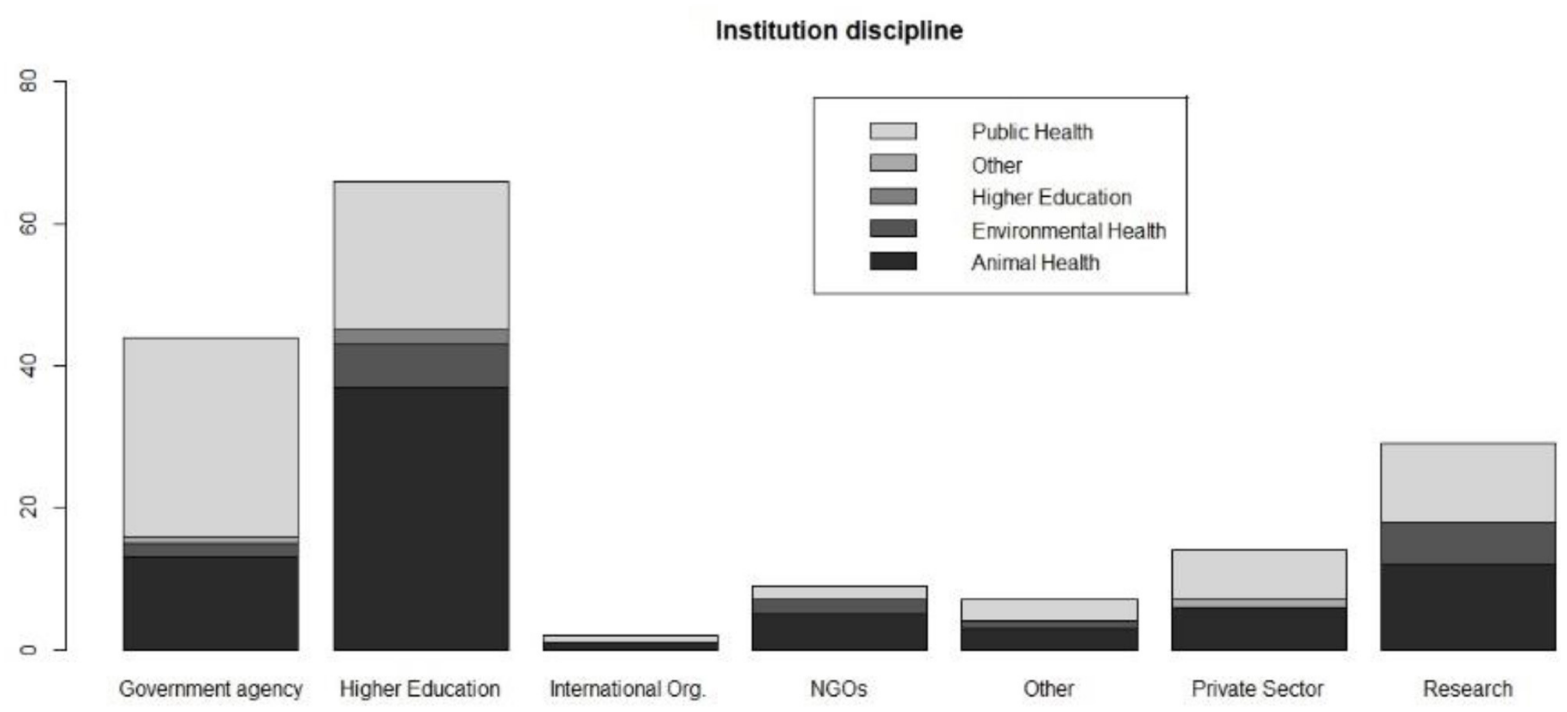
Type of institution and sector where respondents are employed.

The above-mentioned institutions work on Animal Health (*n* = 77; 45.0%), Public Health (*n* = 50; 29.2%), Human Health (*n* = 23; 13.4%) and Environmental Health (*n* = 17; 9.9%). Four respondents did not provide enough details to attribute the discipline of the institution to any of the above categories (hence aggregated under “Other”) (Figure 2).

Most of the respondents stated to be heads/directors (*n* = 57; 33.9%), professors (*n* = 44; 26.1%) and researchers (*n* =18; 10.7%). Thirteen were officers (7.7%) and another 13 respondents were consultants (7.7%). The remaining respondents detailed their positions as medical doctors (*n* = 5; 2.9%), PhD students (*n* = 5; 2.9%), epidemiologists (*n* = 4; 2.3%) and vet clinicians (*n* = 3; 1.7%). Six persons (3.5%) did not answer.

### Questionnaire Section 2—*About one health*

Ninety-nine respondents (57.9%) declared they had heard about One Health, while 36 (21.1%) declared that they had never heard about it; 36 people did not respond to the question.

Respondents were asked to define OH in one sentence. Amongst the 149 answers (87.1% of respondents), only 16 (10.7%) were considered to be a “complete definition.” The “human” component was mentioned by 129 respondents (86.6%), the “animal” component by 110 (73.8%), and the “environmental” one by 63 (42.3%). Fifty-nine respondents (39.6%) included a term among “intersectoral/transdisciplinary/holistic” in the definition, and 34 named “collaboration/sharing” (22.8%).

Considering the disciplinary background of respondents, the best median score for the OH definition (one point for each element) was obtained by “Life Sciences” respondents (3.5, Q1–Q3: 3.25–3.75), followed by “Human Health” respondents (3; Q1–Q3: 3–3). The median score of the other categories (excluding the two respondents with a disciplinary background in Sociology and Engineering, who did not answer) was two. However, the difference in median scores among different disciplinary backgrounds was not significant (Kruskal Wallis rank sum test, *p* = 0.08).

When interviewees were asked if they were currently involved in OH initiatives, the large majority stated to be involved (*n* = 107; 62.6%), while 64 respondents stated that were not. Most people involved had a background in “Animal Sciences” (*n* = 70, 65.4%), followed by “Public Health” (*n* = 16, 15.0%) and “Human Health” (*n* = 12, 11.2%). The involvement significantly differed among disciplinary backgrounds (Fisher Exact test, *p* < 0.01), with 76.1% of the “Animal Sciences” respondents being involved, vs. 55.2% of “Public Health”, 54.5% of “Human Health,” 40% of “Food Safety,” 37.5% of “Environmental Sciences” and 20% of “Life Sciences.” The two respondents with background in Sociology and Engineering declared not to be involved in OH initiatives.

The interviewees were also asked to briefly describe the OH initiatives. Since this was an open answer, it was categorized in: zoonoses (54 answers, 50.4%); OH in general (i.e., a broad comprehensive category that includes activities or initiatives having the scope to promote intersectoral working spaces) (*n* = 12, 11.2%); food hygiene (*n* = 11, 10.2%); antimicrobial resistance (AMR) (*n* = 9, 8.4%); education (*n* = 5, 4.6%); animal health (*n* = 3, 2.8%); human health (*n* = 2, 1.8%); AMR and zoonoses, and AMR and food hygiene (one answer each, 0.9%). Nine interviewees did not specify the characteristics of the OH initiatives they were involved in. OH initiatives on zoonoses were mostly cited by people with a background in “Animal Sciences” (*n* = 36), followed by “Public Health” (*n* = 8) and “Human Health” (*n* = 6). “Environmental Health” respondents were involved in initiatives on zoonoses (*n* = 3), OH in general (*n* = 2) and food safety (*n* = 1). Education activities were cited by “Animal Sciences” respondents only (*n* = 5).

**Figure 3:**
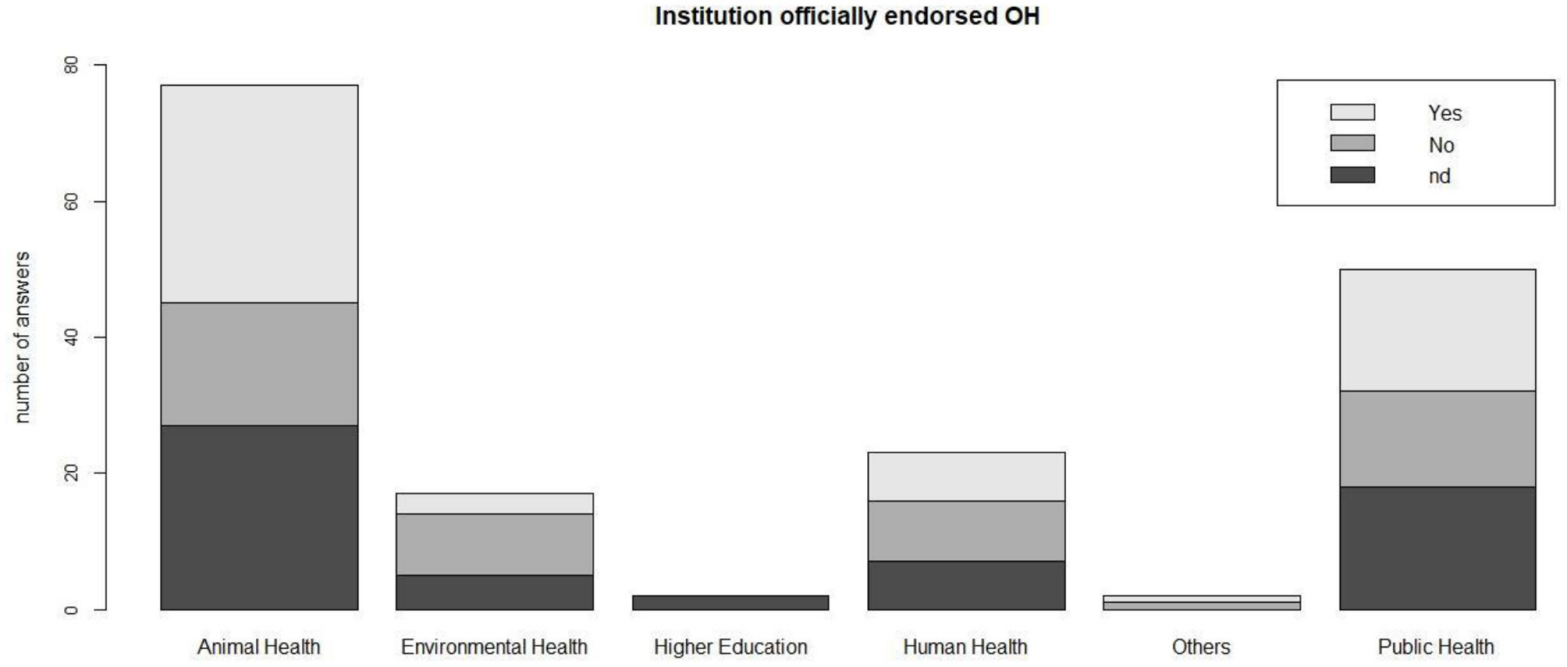
Endorsement of OH initiatives by different types of institutions where respondents are employed.

Sixty-one respondents stated that OH had been officially endorsed by their respective Institutions, while 51 Institutions did not endorse; 59 respondents did not answer. No differences in endorsing or not OH initiatives were found among institution types (Fisher Exact Test, *p* = 0.7) (Figure 3). The institutions reportedly endorsed OH by implementing initiatives regarding education (*n* = 16), OH in general (*n* = 16), animal health (*n* = 7), zoonoses (*n* = 5), AMR, human health, and food safety (*n* = 2 each). Less than half (47.7%) of respondents involved in OH initiatives worked in institutions that officially endorsed OH.

The respondents quoted various examples of programs for which a OH approach was adopted in their institutions. These examples mostly referred to zoonoses surveillance and control (*n* = 24), health education (*n* = 20), AMR (*n* = 17) and food hygiene (*n* = 15). Other cited examples were research (*n* = 9), animal health (*n* = 7), brucellosis and OH in general (*n* = 5), vector-borne diseases (*n* = 4), drawing/writing legislation (*n* = 4), rabies, salmonella, WNV (*n* = 3 each), Campylobacter, Q Fever, HPAI, info sharing and preventive medicine (*n* = 2 each). Air quality, climate change, risk analysis, drinking water quality, human health, mastitis, leishmaniasis, leptospirosis, Lyme disease, rodent-borne diseases, tuberculosis, toxoplasmosis, TSE, and tularemia were cited once.

**Figure 4:**
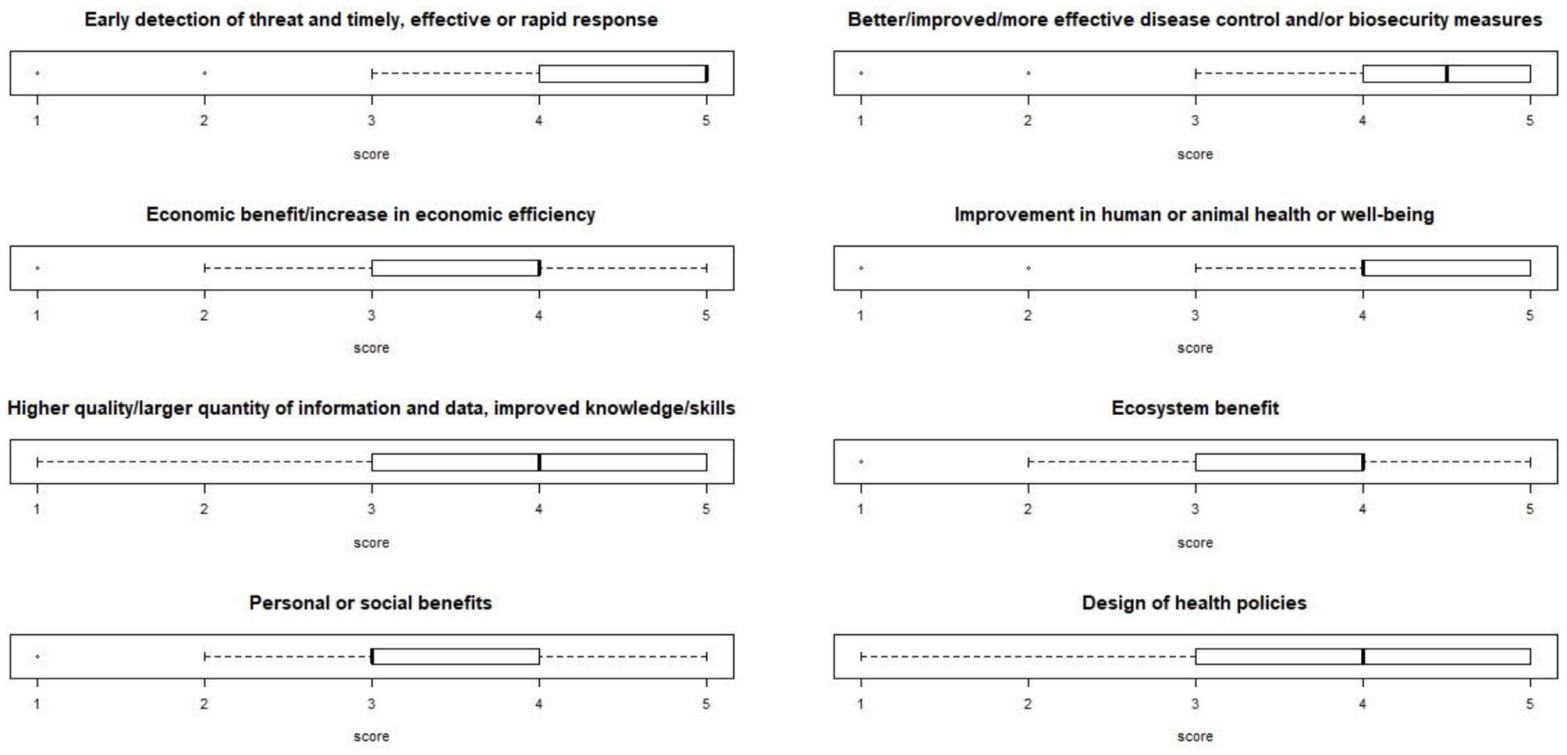
Boxplot of the scoring of the OH advantages, described in literature. Respondents could score each from one (low) to five (high).

When asked to score -from 1 (low) to 5 (high)- some advantages of OH described in literature, “Early detection of threat and timely, effective or rapid response” was considered the major advantage (median score: 5.0), followed by “Better/improved/more effective disease control and/or biosecurity measures” (4.5), thus suggesting that respondents attributed the greatest importance to preventative measures. A lower score was given to “Economic benefit/increase in economic efficiency” (4.0), “Improvement in human or animal health or well-being” (4.0), “Higher quality or larger quantity of information and data and improved knowledge or skills” (4.0), “Ecosystem benefit” (4.0'), “Design of health policies” (4.0) and “Personal or social benefits” (3.0) (Figure 4).

When asked about the existence of boards/committees/associations actively dealing with OH issues/initiatives in their country, 69 respondents answered affirmatively, while 15 stated “no.” Some respondents provided details, stating that such boards mainly deal with AMR, zoonoses, food hygiene, vector-borne diseases and outbreak management.

One hundred fourteen (66.7%) respondents declared that, in their countries, there are formal connections between veterinary/animal health and public health administrations (governmental institutions or services). Conversely, 55 respondents answered “no”; two answers were missing. The answer significantly differed among respondents with different disciplinary backgrounds (Fisher Exact test, *p* = 0.01). The highest “yes” frequency was registered among Food Safety people (all five respondents), Public Health (86.2%), Animal Sciences (69.2%), Human Health (57.1%). “No” answers prevailed among Environmental Sciences (43.8%) and Life Sciences (40%) people, and were given by the two respondents with disciplinary background in Sociology and Engineering.

The respondents were then asked to answer a subset of questions on the level, nature and duration of such cooperation. Most connections were reported to be at national level (*n* = 72 answers, 63.2%), but some respondents indicated national—subnational and local (*n* = 20), national and subnational (*n* = 14), and national and local (*n* = 3) formal connections. Subnational, that is, regional, provincial (*n* = 7), local (*n* = 2) and subnational-local (*n* = 1) connections were also cited. The nature of the cooperation mainly referred to exchange of data (*n* = 93), joint surveillance (*n* = 69), health policies (*n* = 61), joint intervention (*n* = 51), joint preparedness (*n* = 44), joint training (*n* = 41), shared budget (*n* = 9). As regards the length of the cooperation, it ranged from “the last 5 years” (*n* = 23), to “the last 5-to-10 years” (*n* = 30), “10-to-20 years” (*n* = 27), “20-to-30 years” (*n* = 6) and to “more than 30 years” (*n* = 15). This last answer was given by respondents of the following countries: Italy (five respondents), Malta and Hungary (*n* = 2 each), and Belgium, Bosnia-Herzegovina, Denmark, Latvia, Poland, Romania (*n* = 1 each).

Ninety respondents (52.6%) stated they were aware of OH initiatives being implemented in their countries; 14 respondents (8.2%) answered “no” and the others did not answer. According to the respondents, the number of initiatives being implemented varied from 1 to 5 (*n* = 54), from 6 to 10 (*n* = 16), and more than 10 (*n* = 12). The fields of activities concerning these initiatives were: disease surveillance and monitoring (*n* = 80); disease prevention and control (*n* = 74); research (*n* = 70); participants awareness on the programs (*n* = 50); higher education programs (*n* = 41); NEOH and “Knowledge sharing platforms” were cited by one respondent each.

As regards the categories of professionals involved in these initiatives, the following were cited: veterinarians (*n* = 138); medical doctors (*n* = 119); biologists/entomologists (*n* = 82); environmental/ ecosystem specialists/ecologists (*n* = 70); chemists (*n* = 23); sociology/anthropology/gender specialists (*n* = 15); pediatricians (*n* = 15); family doctors (*n* = 13). Other professionals, entered by respondents, were: public health officers (*n* = 3); microbiologists, epidemiologists (*n* = 2 each). Food safety specialists, earth scientists, economists, psychologists, pharmacologists, civil servants/scientific experts, policy risk managers were also quoted.

### Questionnaire Section 3—Zoonotic Diseases, Environmental Health and AMR: Examples of “Burning” OH Issues/Initiatives

Ninety-six people (82.1% of the 117 that answered this question) stated that, in their countries, there is an existing and active cooperation between the Ministry of Health and the Ministry responsible for Animal Health, when dealing with zoonoses. They also stated that there is an obligation to guarantee a reciprocal flux of information or data between Public Health and Veterinary Services. Interviewees were asked to select which zoonotic diseases are controlled and monitored by the Ministry of Health (MoH) and/or Agriculture (MoA). Results are illustrated in Table 1.

**Table 1:** Zoonotic diseases controlled and monitored by the Ministry of Health (MoH) and/or Agriculture (MoA), according to respondents.

**Zoonosis**	**MoA (*n*; %)**	**MoH (*n*; %)**	**MoH + MoA (*n*; %)**
Anthrax	31 (26.5%)	33 (28.2%)	53 (45.3%)
Avian influenza	40 (31.0%)	27 (20.9%)	62 (48.0%)
Brucellosis	40 (32.2%)	22 (17.7%)	62 (50.0%)
Campylobacteriosis	22 (19.4%)	39 (34.5%)	52 (46.0%)
Cysticercosis/taeniasis	39 (39.0%)	26 (26.0%)	35 (35.0%)
Ebola	0 (0%)	84 (82.3%)	18 (17.6%)
Leptospirosis	22 (20.7%)	35 (33.0%)	49 (46.2%)
Plague	6 (7.3%)	49 (59.7%)	27 (32.9%)
Q Fever	28 (26.6%)	26 (24.7%)	51 (48.5%)
Rabies	31 (25.6%)	27 (22.3%)	63 (52.0%)
RVF	19 (25.6%)	26 (35.1%)	29 (39.1%)
Ringworm	17 (25.3%)	26 (38.8%)	24 (35.8%)
Salmonellosis	11 (8.6%)	35 (27.5%)	81 (63.7%)
Toxoplasmosis	15 (14.7%)	38 (37.2%)	49 (48.0%)
Trichinellosis	35 (29.6%)	26 (22.0%)	57 (48.3%)
Tularemia	19 (19.5%)	28 (28.8%)	50 (51.5%)

A couple of questions referred to environmental toxicants. When interviewees were asked about the “level of awareness by the public on zoonoses caused by exposure to environmental toxicants,” scoring from 1 (poor) to five (excellent), the median score attributed by 136 respondents was 2 (Q1–Q3: 2–3). Fifty-nine persons stated they were not competent in the field. The median score given to the “quality of national plans for the prevention and monitoring of toxicant zoonoses” was 3 (Q1–Q3: 2–4). In this case, 85 respondents answered the question, while 88 people stated they were not competent in the field.

**Figure 5:**
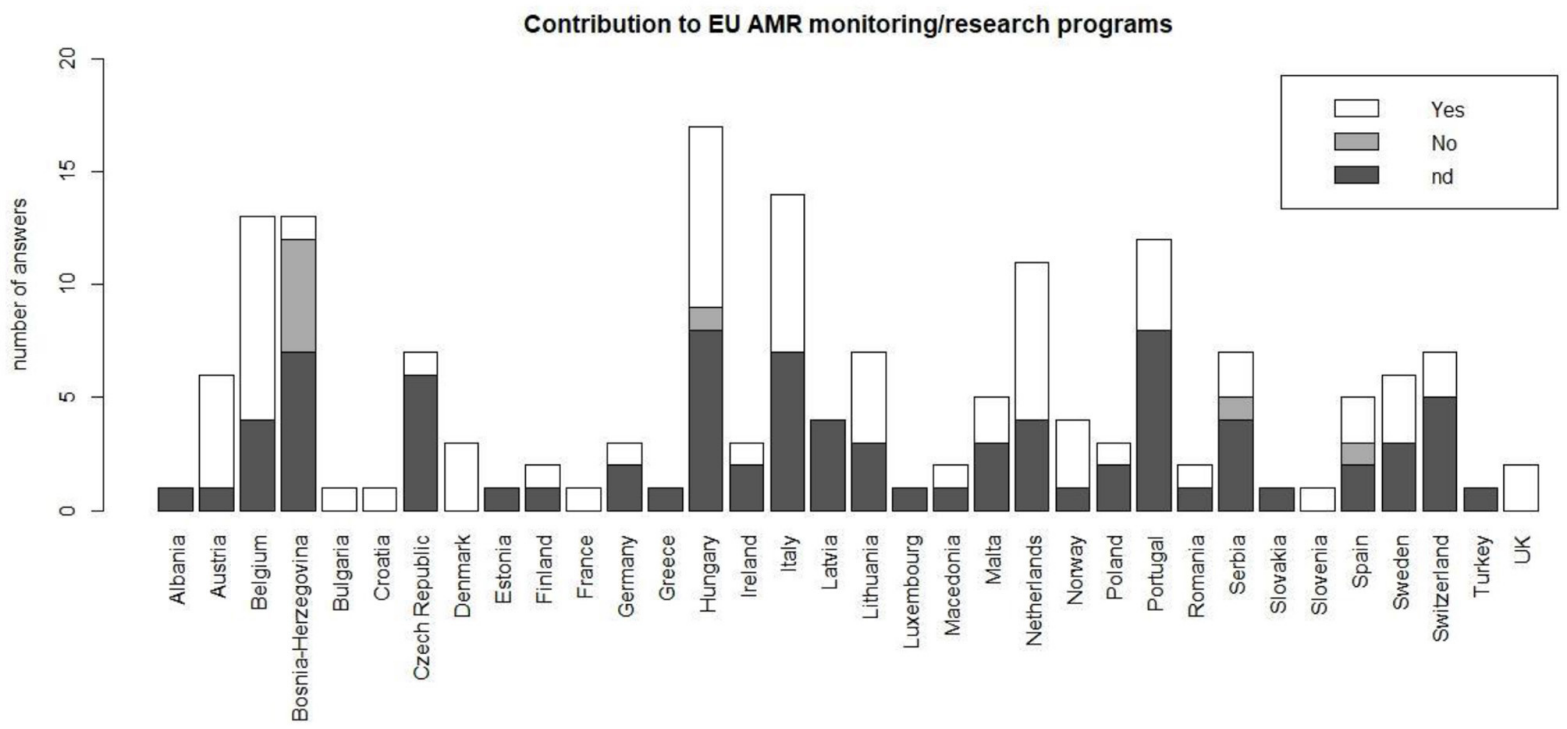
Contribution of respondents' countries to European programs for the monitoring of AMR; nd = not determined/answer not known.

According to 83 respondents (48.5%), their respective countries contribute to the AMR surveillance in Europe, with specific monitoring and research programs. Conversely, 85 people answered “no answer/I don't know.” Conflicting answers within the same country were registered, with eight respondents stating that their respective countries did not contribute to the EU AMR monitoring, although other respondents from the same countries stated they do contribute (Figure 5).

### Questionnaire Section 4—Aspects Limiting Interdisciplinarity/Intersectorality in OH

A “siloed approach” of disciplines was considered a limit for interdisciplinarity and intersectorality by 18.1% of respondents (*n* = 31) working in OH, followed by “lack of resources” (15.8% of respondents, *n* = 27), institutional (10.5%, *n* = 18) and “education limits” (8.2%, *n* = 14). Some people mentioned more than one limit. Two respondents stated that there are no limiting factors (1.2%).

Table 2 describes the level(s) and the opportunities for OH collaborations, according to the interviewees, within professional boards, University Departments, institutions involved in veterinary surveillance and food security, and institutions involved in emergencies management.

**Table 2:** Level and opportunities for OH collaboration at different institutional and/or professional levels.

**Level and opportunities for OH collaboration within**	**Poor (%)**	**Fair (%)**	**Good (%)**	**Excellent (%)**	**n/a (%)**
Professional boards	40 (26.1%)	35 (22.9%)	54 (35.3%)	8 (5.2%)	16 (10.5%)
University Departments	37 (24.3%)	47 (30.9%)	47 (30.9%)	10 (6.6%)	11 (7.2%)
Institutions involved in vet surveillance and food security	18 (11.9%)	46 (30.5%)	64 (42.4%)	13 (8.6%)	10 (11.9%)
Institutions involved in emergencies management	22 (14.5%)	43 (28.3%)	64 (42.1%)	11 (7.2%)	12 (7.9%)
Total	117	171	229	42	49

### Questionnaire Section 5—*Conclusions*

**Figure 6:**
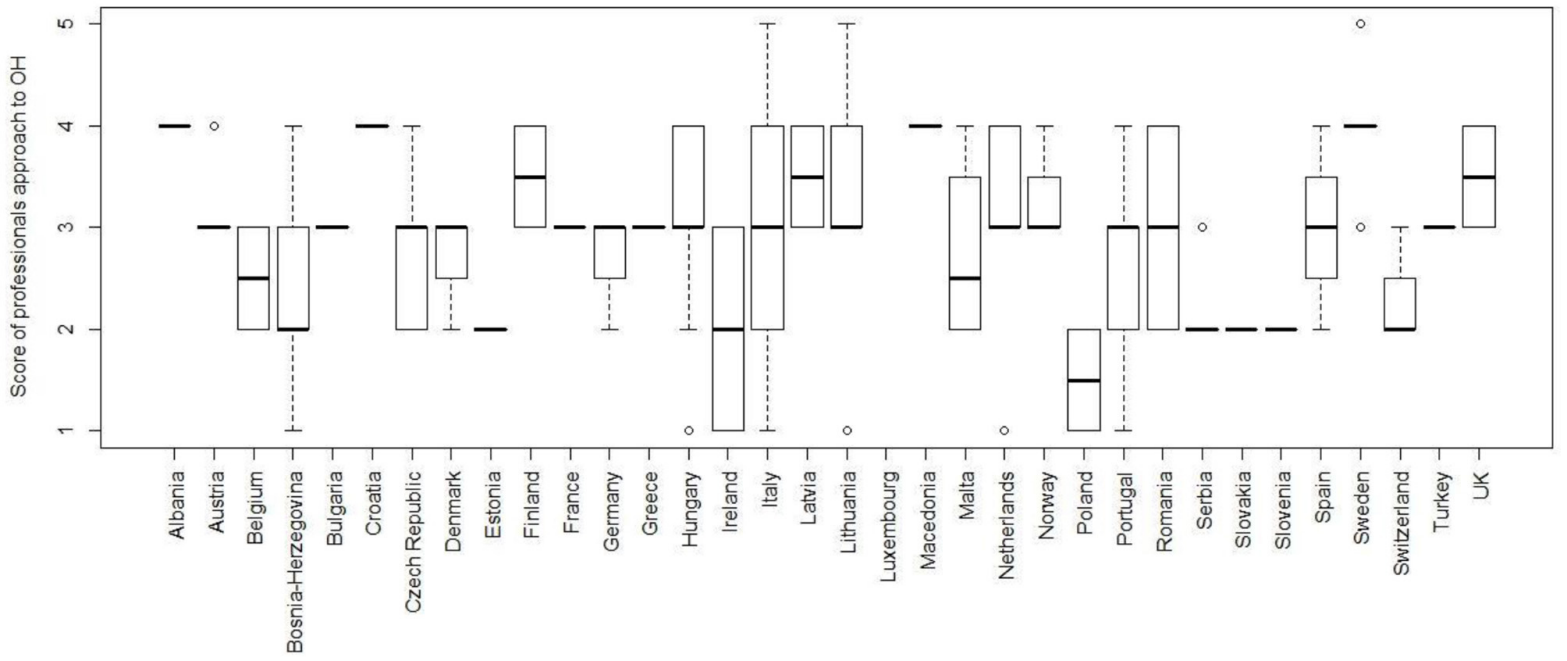
Boxplot of the scores attributed by respondents on the implementation of the OH approach by professionals in their respective countries; scoring from one (poor) to five (excellent).

Respondents were asked to rate how well the OH approach is implemented by the professionals employed/engaged in Veterinary, Public and Environmental Health sectors in their country, scoring from 1 (poor) to 5 (excellent). The median score attributed by 130 respondents was three (Q1–Q3: 2–3). Details of the answers aggregated by countries are illustrated in Figure 6.

The existence of recent formal initiatives to establish and/or to strengthen intersectoral collaboration, aimed at global advocacy of OH approach, was confirmed by 49 respondents (29.8%). Seventeen (10.3%) answered “no” and the other interviewed selected “no answer/I don't know.”

**Figure 7:**
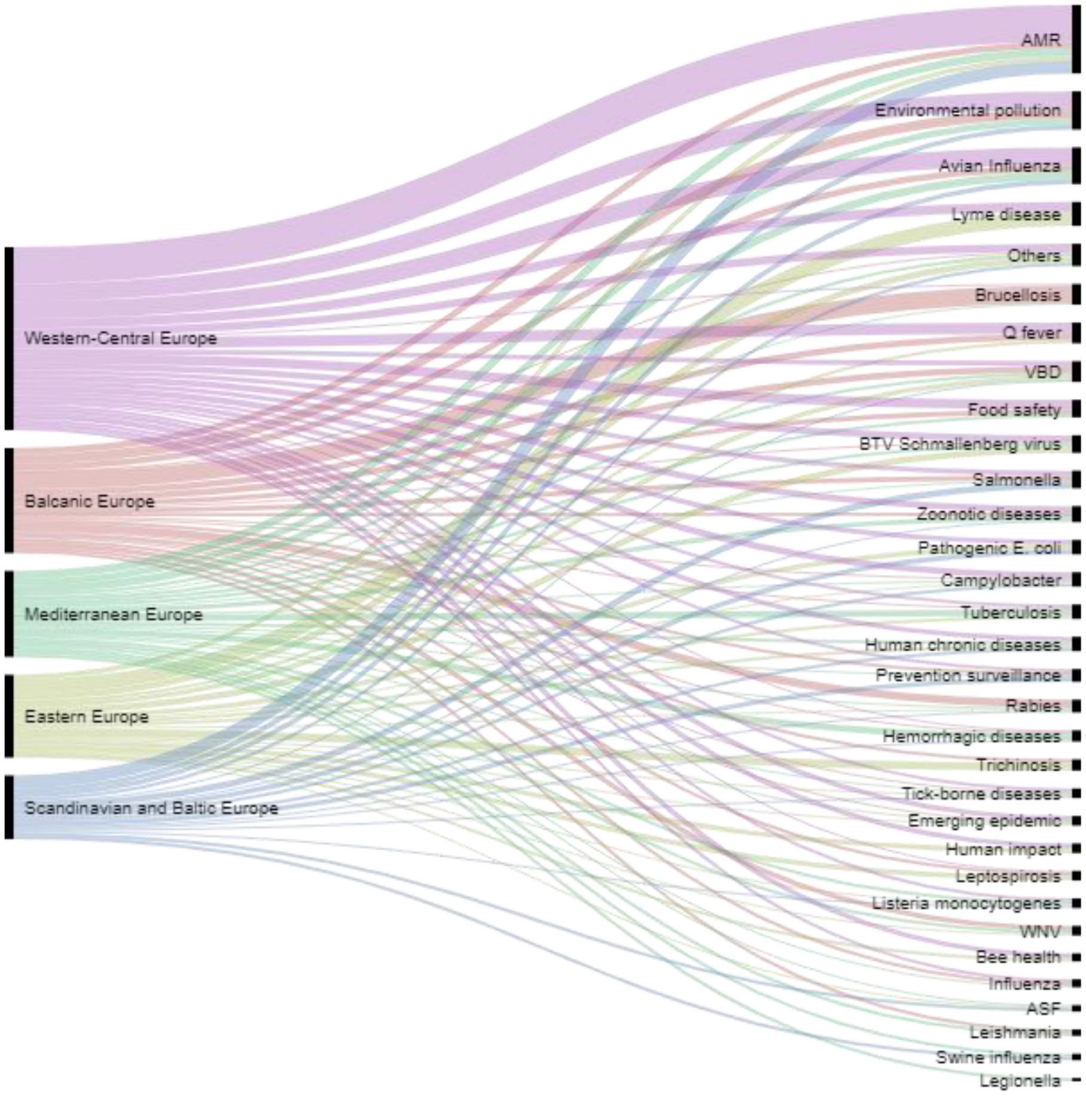
Top environmental, animal and human health issues over the past 5 years cited by countries in the different regional areas.

**Figure 8:**
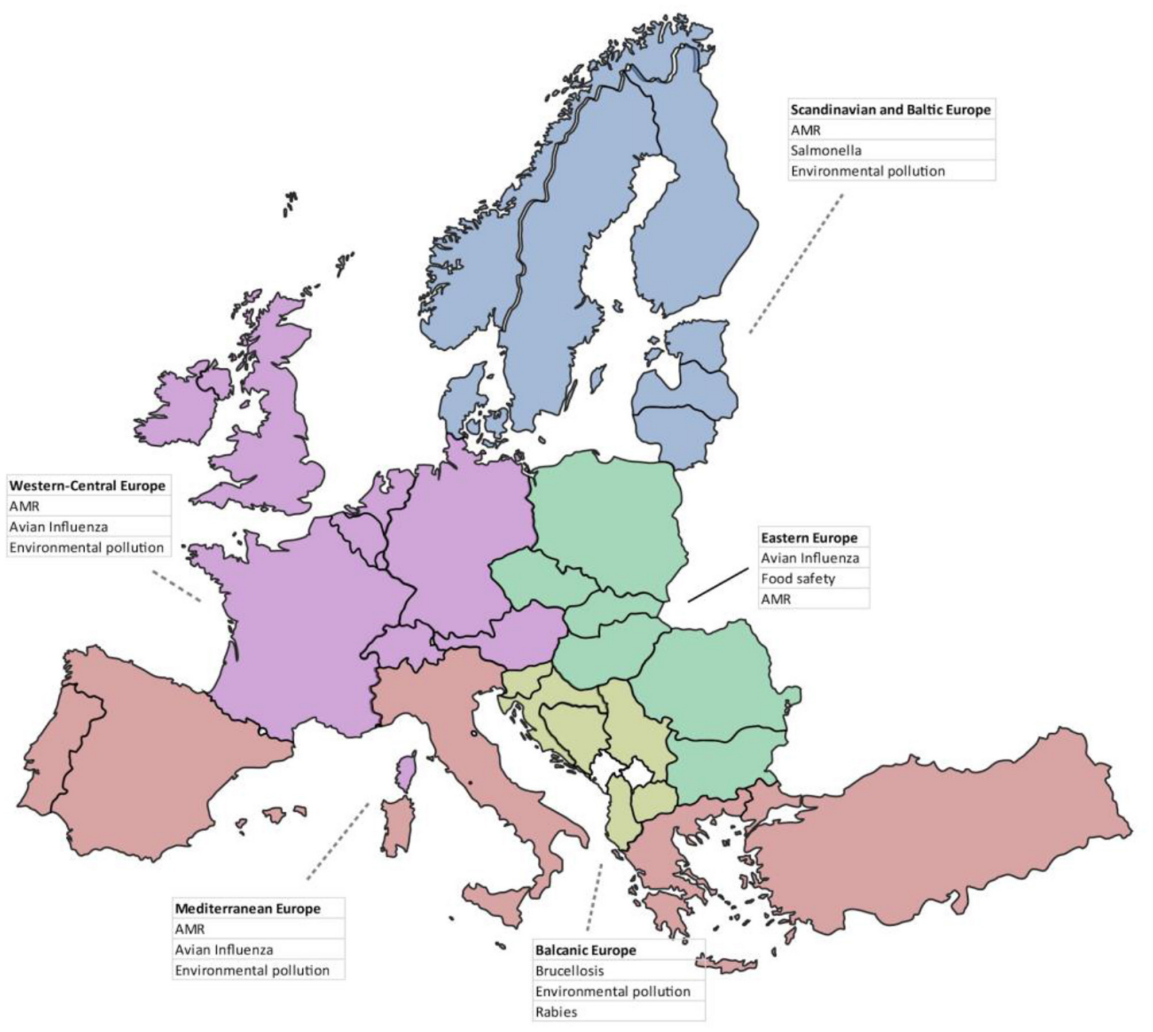
Top three environmental, animal and human health issues over the past 5 years cited by respondents, divided by geographical regions.

When asked to cite the top three environmental, animal and human health issues in their country over the past 5 years, most respondents cited AMR (*n* = 44 respondents; 25.7%), avian influenza (*n* = 32; 18.7%) and environmental pollution (*n* = 27; 15.8%). A Sankey diagram shows all answers by regional areas (Figure 7). When considering answers by group of countries in the same geographical region, we observed differences in the top three issues (Figure 8): AMR was cited in all regions except from Balkan countries, avian influenza in all regions except from Balkan and Scandinavian/Baltic countries, and all except Eastern European countries cited environmental pollution.

Strictly related to section 4, respondents were asked to answer where and what are the gaps in OH schemes. Answers mostly referred to the approach of institutions (*n* = 24), followed by problems of funding (*n* = 12) and communication (*n* = 11), the siloed approach of disciplines (*n* = 11), and scarce education (*n* = 10) and awareness (*n* = 8).

**Figure 9:**
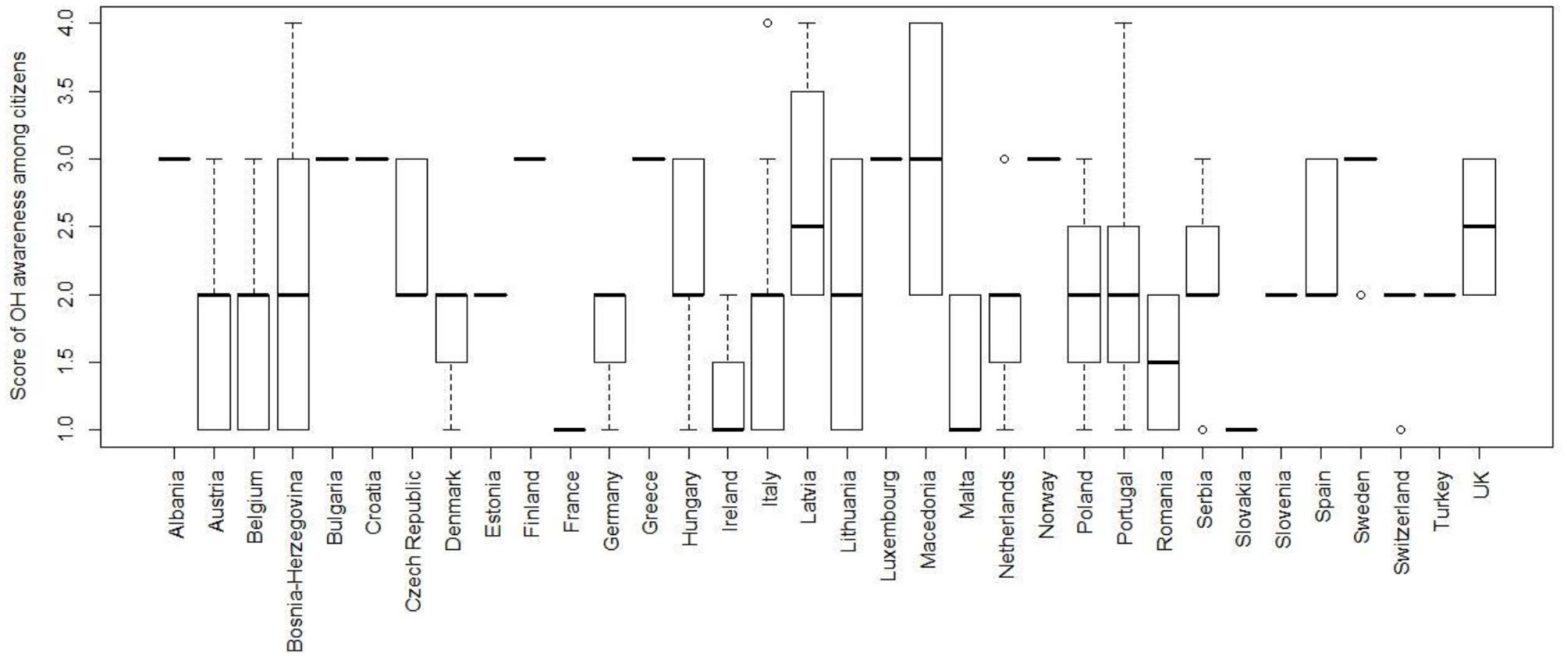
Boxplot of the scores attributed by respondents to the level of awareness on OH in citizens in their respective countries; scoring from one (poor) to four (excellent).

According to respondents, the level of awareness/perception of OH amongst citizens/consumers their country is not high. Indeed, they could score from one (poor) to four (excellent), and the median score was 2.0 (Q1–Q3: 1.75–3.0). Details of the answers aggregated by countries are illustrated in Figure 9.

### Questionnaire Section 6—*End of questionnaire*, Including Comments/Remarks/Suggestions

Only some respondents contributed to this last section, by adding a few comments, remarks and suggestions: four participants suggested that the questionnaire should have been less lengthy and more essential, with less detailed and clearer questions; three signaled that some questions and/or definition were not very clear (i.e., environmental toxicants and toxicant zoonoses); other three suggested that all questions should have the option “don't know/not sure about” (actually “don't know” was always available whenever applicable); a couple of respondents suggested that the text should have been revised by a English native speaker; two stated that the questionnaire was OK and no remarks/comments were needed; one reported that the questionnaire could not be saved in the process of filling in.

A few respondents included a set of comments/suggestions which deserve to be reported here […“*In view of the siloed mentality prevailing in the country, it is difficult to ascertain that the knowledge expressed in this questionnaire captures all the activities happening in other Ministries.”*, …. “*the actions in favor of OH will have to continue their creative work in the Universities, both at undergraduate and graduate levels*”, …”*OH issues have to be emphasized at Academic level in each of the 3 OH disciplines/components* (i.e., *Animal/Human-Public/Environmental Health)”*, … “*include requirements for OH in health sector legislation, including specific budgeting of resources*..”, …“*Having projects managed by people having knowledge of more than one discipline and who are open to listen to all collaborators*.”] and to be recalled/quoted in the discussion and conclusions.

## Discussion

Our survey aimed to provide an overview of the existence and the implementation of OH actions/initiatives in Europe and neighboring areas, of the type of institutions and disciplines involved in such initiatives, and on current limits to OH implementation according to professionals working in relevant fields (e.g., education, research, government).

Overall, the response rate (51.3%) of our questionnaire survey falls within the range of the response rates of similar online questionnaires (22). In a study on response rate in organizational research, (23) reported an average response rate of 52.7% for studies that utilized data collected from individuals, while the average response rate for studies that utilized data collected from organizations was 35.7%. However, few countries reached or exceeded the expected minimum number of questionnaires answered (nine per country).

The questionnaire design and distribution were managed such to reach an equal number of respondents from each of the three OH pillars/components, that is, animal health, human-public health and environmental health, evenly across countries, types of institutions and disciplines. However, there was a larger proportion of respondents from animal health, thereby skewing answers toward human and animal health with less consideration of ecosystem health and on collaborative and system aspects of OH. This may be explained by the fact that the OH approach and the OH movement have been promoted strongly by animal health and public health, as documented in some of the most accredited and comprehensive reviews on the origin and development of OH (7, 8). In addition, the larger number of respondents with a professional background in animal health/animal sciences, as well as the larger number of academics/researchers, seem to reflect the composition of NEOH and EurNegVec networks, who implemented the survey. As COST Actions are often research/academic networks, MC members and MC substitutes likely were better connected to academic and research colleagues than officials at Ministries/Directorates. The underrepresentation of nature/environment-related human health benefits and ecosystem health could be a limitation of this study and at the same time a reflection of the origins of OH and the networks of the participants of the two COST Actions. Potentially, more respondents from ecosystem fields could have been engaged with stronger wording around Ecohealth.

Another limitation of our study is that data were collected in 2016–7 and therefore may not represent today's OH landscape in the countries surveyed. Nonetheless, it provides a baseline against which future studies of this type can be compared thereby contributing to documentation of how OH evolves in Europe and neighboring areas.

Although One Health was a familiar concept for the majority of respondents, only few were able to provide comprehensive definitions. Their answers demonstrated a traditional understanding of OH evolving around the linkages between “human” and “animal” health. The environmental health component was mentioned less frequently, which suggests that the three pillars/components of OH are overall not perceived as having equal importance. Similar differences amongst the three OH components seem to emerge from other questions, for example most of the respondents, who declared to be presently involved in OH initiatives, had a background in “Animal Sciences.” Indeed, we registered a greater involvement in OH initiatives among Animal Sciences professionals, with 76.1% of respondents in this category being involved, vs. around half of respondents in “Public Health” and “Human Health” categories, and 37.5% of “Environmental Sciences.”

Only slightly more than half of the respondents involved in OH initiatives seemed to be personally engaged in OH. Although adopting a OH approach for many is still a matter of a personal attitude and less of institutionalization, it seems that some steps toward official endorsement of OH are being taken, and this may constitute a move toward institutionalization of OH—at least in the human and animal health fields. Another challenge emerging from our results is the low public awareness on OH. This could be addressed through extension and information campaigns to give the general public access to OH; and actually, such activities are being started. Existing OH initiatives mentioned by respondents related mainly to “classic zoonoses” (prevention, control, surveillance). This is probably because the majority of respondents belonged to institutions working in the animal and public health disciplines, and these disciplines were those that mainly endorse OH. Other disciplinary sectors were less represented in our sample, so environmental problems, such as climate change, pollution, toxicants, socio-environmental aspects, were less mentioned.

In addition, we hypothesize that “chronic” environmental issues (i.e., contaminated agriculture soil and/or fallow land) are less visible and receive less attention both from the general public and health authorities/policy makers. Moreover, such environmental problems are usually more difficult to control and prevent.

The OH approach was adopted for zoonoses (surveillance and control), health education, AMR and food hygiene. Coincidentally, these are also common areas of the field of Veterinary Public Health and this brings up the question whether respondents differentiated between the two. Veterinary Public Health (i.e., the contribution of veterinary medicine to public health) has indeed gradually evolved in OH and in “ecosystem health”—that includes the whole ecosystem, and that considers health and ecosystems and their relevance for global health development (7).

According to the respondents, the most important advantages of OH are preventative measures such as early detection/rapid response, and effective disease control and/or biosecurity measures which actually put “prevention” at the highest rank. Lower scores were attributed to health improvement, knowledge/skills improvements, ecosystem and personal/social benefits, thus confirming a more “traditional” approach oriented toward disease surveillance and response, also suggesting a poor understanding of the environmental health component and still an apparent existing dichotomy between OH and Ecohealth. The fact that two thirds of respondents reported formal connections between veterinary and public health administrations (governmental institutions or services) is noteworthy. Remarkably, the highest “yes” frequency was registered among those respondents involved in Food Safety, Animal and Public/Human Health, while “No” answers prevailed amongst Environmental and Life Sciences persons, suggesting that respondents involved in the former disciplines seem to be more aware of such formal connections. Other respondents may be more aware of Ecohealth connections.

As pointed out by some authors (24–27), the two approaches, despite their apparent dichotomy, have a common ground and unifiying attributes. In fact, according to Zinsstag (7) both Ecohealth and One Health movements emphasize a holistic understanding of health beyond the purely biomedical and champion system thinking as a way of achieving a greater understanding of health problems; both approaches espouse inter- and trans-disciplinary research and collaborative participation. Literature in the field shows that the concepts continue to evolve and be discussed. For example, Lackey (28) promoted a challenging discussion on the values of ecosystem health, Antoine-Mussiaux et al. (29) provided an analysis on framing “nature” or the environment within the scientific communities involved, Harrison et al. (30) addressed the calls for convergence between OH and Ecohealth. Some areas already well-developed in Ecohealth (e.g., relationships between health and ecosystems or between health and sustainable development) have been growing in importance within the One Health movement. Each approach has its own strengths and -by working together- a greater impact in global health and sustainability may be achieved (25). In line with this view, NEOH has therefore established a new Network for Ecohealth and OH as a chapter of the Ecohealth International organization (https://www.ecohealthinternational.org/regional-chapters/europe/).

Section 3, regarding zoonotic diseases, environmental health and AMR, provided interesting insights. More than 80% of the respondents reported an active cooperation in their countries between the Ministry of Health and the Ministry responsible for animal health, when dealing with zoonoses, also stating that there is an obligation to guarantee a reciprocal flux of information between Public Health and Veterinary Services. When respondents were asked to select which zoonotic diseases (from a list taken from Public Health England website) are or should be monitored and controlled by MoH and/or MoA, there was a convergence –with a few exceptions, that is, Ebola, ringworms, cysticercosis/taeniasis- in attributing the responsibilities to both Ministries. This could be related to the different organization of the Health System and Veterinary Services in the target countries.

Two questions were asked about environmental toxicants and it was requested to score the level of awareness by the public on “toxicant zoonoses” (zoonoses caused by exposure to environmental toxicants) and the quality of national plans for their prevention/monitoring. The percentage of respondents to these questions was not satisfactory; in fact, more than half of them stated that they were not competent in the field, thus suggesting that “non-traditional zoonoses” are generally less known or less taken into consideration.

While nearly half of the interviewees stated that their respective countries contribute to EU AMR surveillance with specific monitoring and research programs, there were some conflicting answers amongst respondents, that is, within the same country, some respondents stated that their country contribute to the EU AMR monitoring and others stated the contrary. However, the European surveillance systems/networks [e.g., EARS-Net (31)] and the international guidelines (16) are likely helping the full implementation of the National Action Plans (NAPs) to fight AMR.

In the section regarding the aspects limiting interdisciplinarity and intersectorality in OH (section 4), the “siloed approach” of disciplines was the most commonly mentioned limiting factor. Lack of resources, institutional and education limits were also cited. All these factors have long been recognized as barriers to moving toward OH (32).

When asked to score the level and opportunities for OH collaboration at different institutional and/or professional levels, the higher proportion of “good” and “excellent” scores were attributed to institutions involved in animal health surveillance/food security, and institutions involved in emergencies management. “Poor” scores prevailed as regards professional boards and university departments, but were attributed by a maximum of 26% of respondents, suggesting that in COST countries there is an overall positive perception about level and the opportunities for OH collaboration.

When asked to rate (one being “poor,” and five “excellent”) how well the OH approach is implemented by the professionals employed/engaged in Veterinary, Public and Environmental Health sectors, respondents gave a median score of 3 (Q1–3: 2–3). Although the great difference in the number of respondents per country makes it difficult to compare and comment the results, the authors consider such overall score (3) not much “encouraging.” Professionals still need to do more efforts for implementing the OH approach in their respective countries. Alike, the answers on the existence of initiatives to establish/strengthen intersectoral collaboration, aimed at global advocacy of OH approach, yielded a not much promising picture: in fact, only 30% of the respondents are aware of such initiatives.

When asked to cite the top three environmental, animal and human health issues in their country over the past 5 years, most respondents cited AMR, avian influenza and environmental pollution. Such results seem consistent with the epidemiological situation at the time the questionnaire was circulated (2016–17). Indeed, in the period under review, AMR was –and actually still it is- capturing most attention by the international scientific community. Zoonotic avian flu was quoted as the second most important health topic as, since 2013, thousands of human cases and many deaths have been reported worldwide. Environmental pollution is considered by the respondents a “chronic” problem, quite often difficult to remediate and to solve, which induce several acute and long-term effects on human health and ecosystems.

The authors believe that if the questionnaire were to be administered nowadays, the top health issue would have certainly been the recent (and ongoing) COVID-19 pandemic.

Regional differences could also be observed, in particular between countries belonging to Balkan Europe and the other groups of countries: this is the only group where AMR was not included among the first three OH issues, while brucellosis and rabies were at the first and third position respectively. This reflects the endemic situation and the challenge posed to public health by these diseases in the Balkan countries (33–36). It is noteworthy that *Salmonella* falls in the top three issues in the Scandinavian and Baltic Europe: this foodborne pathogen, which has been the object of a strong and successful surveillance and control programme in these countries (37), is nonetheless considered as a top priority when public health is involved.

In this section, another important question/issue was about the level of awareness/perception of OH amongst citizens/consumers: the median score attributed by the respondents in their respective countries was only 2, and such overall data is not much “encouraging.” This result not only highlights that respondents know that great work still needs to be done to raise public awareness about OH, but also that we all, as members of research and academic institutions, have failed to engage citizens sufficiently. Though there were differences in the number of respondents per country, thus making it difficult to comment on the results, it is certainly crucial that academic and research Institutions, ministries, professional boards have to improve on dissemination, information and education activities, through public engagement and promotion activities on OH for general public and stakeholders.

The gaps on OH schemes cited were strictly related to the question posed in section 4 about factors limiting OH based on the respondents' personal experience. Although many professionals seem highly motivated to endorse the OH approach, barriers exist especially at the institutional level. In fact, interviewees underlined the lack of OH approach in institutions. The “siloed approach” of disciplines, scarce funding and education/awareness limits are other limits to intersectorality, resulting in poor networking, differing priorities and a lack of understanding between sectors.

Respondents from different sectors preferentially selected (prioritized) their own directorates/ministries when looking at the control and monitoring of zoonotic diseases, while actually in most countries, both MoH and MoA are engaged. In Italy, where Veterinary Services are under the Ministry of Health, such differences tend to be less evident.

Despite the limitations described, this study provides an overview of perception and experiences in OH and OH initiatives in Europe, putting into evidence major gaps and challenges as well as opportunities to better apply OH approach. Identifying how initiatives are currently working and knowing their promoting and hindering factors has allowed insights into how improved education and incentives for those working in different areas of the field could change efficiency/effectiveness of OH implementation in years to come. As regards education, academia can play an important role to develop and implement a common OH curriculum to be used by the different European Universities to teach OH approach, strategies, and methods. Health and Environment Institutions/Ministries –together with the Academia, the professional boards, and NGOs- can promote awareness campaigns on the importance of OH approach with stakeholders and the general public at large. Scientists should make efforts to better communicate and share their research results on OH issues to the public and to politicians and policy-makers: such process of sharing information and scientific-based opinions/recommendation would likely make scientists more listened to. The present COVID-19 pandemic –with its enormous toll of victims and socio-economic consequences- has probably greatly contributed to a better awareness of the importance of OH, and -even more- Planetary Health.

Stakeholders, professional boards, the research world and academia, together with citizens must capitalize the momentum gained and use it as a trigger with politicians and decision makers –nationally and internationally to advance OH and related approaches.

## Data Availability Statement

The raw data supporting the conclusions of this article will be made available by the authors, without undue reservation.

## Ethics Statement

An informed consent form was provided at the beginning of the questionnaire where the participants were advised that the questionnaire was anonymous and that, by completing and submitting it, they voluntarily agreed to participate. Ethical approval was sought and granted by the Clinical Research and Ethical Review Board at the Royal Veterinary College, grant holder of COST Action TD1404 NEOH (ref. prot. n. URN 2016 1554).

## Author Contributions

FC: conceptualization and design of the questionnaire survey, data cleaning and managing questionnaire answers, creation of figures and tables, drafting and editing manuscript. LT: data cleaning and managing questionnaire answers, data management and analysis, creation of figures and tables, drafting and editing manuscript. SS: conceptualization and design of the questionnaire survey, collaboration in the survey distribution, drafting and editing manuscript. AB: data cleaning and managing questionnaire answers, collaboration in data analysis, collaboration in drafting manuscript. AM: general contribution in the study design, general supervision as EURNEGVEC chair, collaboration in the survey distribution. DM: general contribution in the study design, collaboration in the survey distribution. BH: conceptualization and design of the questionnaire survey, general supervision as NEOH chair, drafting and editing manuscript. DDM: conceptualization and design of the questionnaire survey, general coordination and supervision of the questionnaire distribution, managing questionnaire answers, drafting and editing manuscript. All authors: reviewing the final version of the manuscript.

## Conflict of Interest

The authors declare that the research was conducted in the absence of any commercial or financial relationships that could be construed as a potential conflict of interest.
