# Supplementary material for: A Survey on One Health Perception and Experiences in Europe and Neighboring Areas
Source: Front Public Health. 2021 Feb 17;9:609949. doi: 10.3389/fpubh.2021.609949 (PMC7925822; doi:10.3389/fpubh.2021.609949)
Supplement: Supplementary file 1 [file Data_Sheet_1.PDF]

- SCOPE OF THE QUESTIONNAIRE AND DETAILS –
This survey has been promoted under the initiatives of the COST Actions TD1404 “Network of Evaluation of One Health-NEOH” and TD1303 "European Network for Neglected Vectors and Vector-Borne Infections - EURNEGVEC".

The purpose of this questionnaire is to collect information and data on the existence/ implementation of One Health (OH) actions/initiatives in your country. Besides collecting information on existing or planned OH initiatives in European countries (i.e. COST member countries), the questionnaire will also be used to get an overview on broader OH aspects in non-EU countries / third countries.

In order to have a representative survey, the OH questionnaire shall be answered by key respondents representing the three OH components, Animal Health, Human Health/Public Health and Environmental Health, represented by the following main sub-sets of respondents:

- public institutions/ministries
- academia/research
- private sector
- NGOs and other stakeholders

We recommend that you only answer the questions covered by your expertise.

The survey should not take longer than 10-15 min to complete.

- DISCLAIMER -
i. By completing and submitting, you are voluntarily agreeing to participate.
ii. Your responses will remain anonymous. No one will be able to identify you or your answers, and no one will know whether or not you participated in the study.
iii. The information collected may not benefit you directly, but the information learned in this study should provide more general benefits for the research purposes of the project.
iv. Your answers will be sent to a link at https://docs.google.com where data will be stored in a password protected electronic format. docs.google.com does not collect identifying information such as your name, email address, or IP address.

Please, be so kind to complete the questionnaire by end of March, 2017.
Thank you in advance for your kind collaboration and support.

The NEOH WG2 and EURNEGVEC WG1 team

-ACKNOWLEDGEMENT-
Supported under COST Action TD1404 (NEOH) and TD1303 (EURNEGVEC), supported by COST (European Cooperation in Science and Technology)

http://www.cost.eu/COST_Actions/tdp/TD1404?parties
http://www.eurnegvec.org/
http://www.cost.eu/COST_Actions/bmbs/TD1303?parties.

For any query concerning the questionnaire, you may get in touch with Sara Savić (sara@niv.ns.ac.rs), Daniele De Meneghi (daniele.demeneghi@unito.it), Francesco Chiesa (francesco.chiesa@unito.it).

One Health Questionnaire

*Mandatory fields

1. GENERAL INFORMATION

QUESTION 1.1

Which is your country of work? *

Choose

QUESTION 1.2

What is your disciplinary background? *

- Environmental Sciences
- Human Health
- Animal Sciences
- Public health
- Altro:

Please, specify your University degree title

Your answer

QUESTION 1.3

At which type of institution are you employed? *

Choose

*

Discipline of the institution

- Environmental health
- Animal sciences
- Human Health
- Public health
- Altro:

What is your present position?

Your answer

2. ABOUT "ONE HEALTH"

QUESTION 2.1

Are you a member of any of the following networks/projects?

- ☐ NEOH
- ☐ EURNEGVEC
- ☐ other ‘One health’ (OH) networks/projects

if "other", please, specify

Your answer

If not, have you ever heard about OH?

- ○ Yes
- ○ No

QUESTION 2.2

What is your understanding of OH?

Please, define OH in one sentence

Your answer

QUESTION 2.3

Are you currently involved in OH initiatives ? (e.g. surveillance, prevention and control of: vector-borne zoonoses; environmental contaminants in food; rabies; etc)

- Yes
- No

If yes, please give a brief description of the initiatives

Your answer

QUESTION 2.4

Has your institution officially adopted/endorsed OH?

- Yes
- No
- No answer/I don't know

If yes, describe how

Your answer

QUESTION 2.5

Please list programs/activities in which your institution follows a OH approach, if any

Your answer

QUESTION 2.6

How relevant are the following OH advantages described in the literature approach?

Grade from 1 (not relevant) to 5 (highly relevant)

	1	2	3	4	5
Early detection of threat and timely, effective or rapid response	◯	◯	◯	◯	◯
Better/improved/more effective disease control and/or biosecurity measures	◯	◯	◯	◯	◯
Economic benefit/increase in economic efficiency	◯	◯	◯	◯	◯
Improvement in human or animal health or well-being	◯	◯	◯	◯	◯
Higher quality or larger quantity of information and data and improved knowledge or skills	◯	◯	◯	◯	◯
Ecosystem benefit	◯	◯	◯	◯	◯
Personal or social benefits	◯	◯	◯	◯	◯
Design of health policies	◯	◯	◯	◯	◯

Please, list any additional advantage:

Your answer

QUESTION 2.7

Are there boards/committees/associations actively dealing with OH issues/inititatives in your country?

- Yes
- No
- No answer/I don't know

If yes, please provide detail the initiative(s), participants and the issues discussed

Your answer

QUESTION 2.8

Do you know if there are FORMAL connections between veterinary/animal health and public health administration in your country (governamental institutions or services)?

- Yes
- No

If yes, please explain the authority, level, responsibilities and title of the existing body

Your answer

At which level ?

- National
- Sub-national (i.e. regional, provincial)
- Local

Nature of cooperation

- [ ] exchange of data
- [ ] joint surveillance
- [ ] shared budget
- [ ] joint intervention
- [ ] joint preparedness
- [ ] joint training
- [ ] health policies
- [ ] other:

Formal cooperation (if any) established in

- ○ last 5 years
- ○ last 5-10 years
- ○ last 10-20 years
- ○ last 20-30 years
- ○ More than 30 years

QUESTION 2.9

To your best knowledge and your personal opinion, are there any OH initiatives being implemented in your country?

- ○ Yes
- ○ No
- ○ No answer/I don't know

If yes, please indicate how many

- ◯ 1-5
- ◯ 6-10
- ◯ >10

please tick the fields of activities where these initiatives are implemented

- ☐ Disease surveillance and monitoring
- ☐ Disease prevention and control
- ☐ Awareness of participants of the programs
- ☐ Higher Education programs
- ☐ Research
- ☐ other:

What are the specific topics covered by these initiatives? Please provide the list (preferably with web links) of OH based initiatives which you refer to.

Your answer

IF NOT, please detail the fields of activities where you think these programs should be implemented

- ☐ Disease surveillance and monitoring
- ☐ Disease prevention and control
- ☐ Awareness of participants of the programs
- ☐ Higher Education programs
- ☐ Research
- ☐ other:

QUESTION 2.10

To your knowledge, which of the following professionals are directly employed/engaged in OH initiatives in your country?

- ☐ Veterinarians
- ☐ Medical doctors
- ☐ Pediatricians
- ☐ Family doctors
- ☐ Biologists/entomologists
- ☐ Chemists
- ☐ Environmental/ecosystem specialists (ecologists)
- ☐ Sociology/anthropology/gender specialists
- ☐ No answer/I don't know
- ☐ other:

3. ZOONOTIC DISEASES, ENVIRONMENTAL HEALTH AND ANTIMICROBIAL RESISTANCE: EXAMPLES OF (BURNING) OH ISSUES

QUESTION 3.1

Is there an existing and active cooperation between ministry of health and ministry responsible for veterinary medicine when dealing with zoonoses?

- Yes
- No
- No answer/I don't know

If yes, please give an example

Your answer

QUESTION 3.2

Is there a legislative norm / rule regulating the collaboration between Public Health and Veterinary services (obligation to guarantee a reciprocal flux of information/data)?

- Yes
- No
- No answer/I don't know

If yes, please describe

Your answer

QUESTION 3.3

Select among the listed zoonotic diseases which are controlled and monitored by the Ministry of Health and/or Agriculture

List based on: Zoonoses and the Human-Animal-Ecosystems Interface (http://www.who.int/zoonoses/en/)

	Ministry of Health	Ministry of Agriculture	Both
Anthrax	○	○	○
Avian influenza	○	○	○
Brucellosis	○	○	○
Campylobacter	○	○	○
Cysticercosis	○	○	○
Ebola, Crimean-Congo HF, Lassa and Marburg viruses	○	○	○
Leptospirosis	○	○	○
Plague	○	○	○
Q fever	○	○	○
Rabies	○	○	○
Rift Valley fever	○	○	○
Ringworm	○	○	○
Salmonella	○	○	○
Toxoplasmosis	○	○	○
Trichinellosis	○	○	○
Tularemia	○	○	○

QUESTION 3.4

What is the level of awareness by the public on zoonoses caused by exposure to environmental toxicants?

	1	2	3	4	5	
poor/no awareness	○	○	○	○	○	excellent awareness

○ not competent in this field

QUESTION 3.5

What is the quality of national plans for toxicant zoonoses prevention and monitoring?

	1	2	3	4	5	
poor	○	○	○	○	○	excellent

○ not competent in this field

QUESTION 3.6

Does your country contribute to the EU AMR (antimicrobial resistance) monitoring with specific monitoring and research programme (e.g. DANMAP)?

- ◯ Yes
- ◯ No
- ◯ No answer/I don't know

If yes, please describe

Your answer

One Health Questionnaire

4. ASPECTS LIMITING INTERDISCIPLINARITY/INTERSECTORIALITY

QUESTION 4.1

If you have worked in OH in your country, which have been the factors limiting the interdisciplinarity/ intersectorality?

Your answer

QUESTION 4.2

Describe level and opportunities for collaborations in your country within:

	N/A	Poor	Fair	Good	Excellent
Professional boards (with special attention to those between veterinarians and medical doctors)	○	○	○	○	○
University courses (with special attention to those between veterinary sciences and medical sciences).	○	○	○	○	○
Institutions (ministries and/or services) regulating/managing veterinary surveillance and control of food chains	○	○	○	○	○
Institutions (ministries and/or services) responsible for emergencies, including disaster management, rehabilitation and resilience	○	○	○	○	○

5. CONCLUSIONS

QUESTION 5.1

Rate how well the OH approach is implemented by professionals employed/engaged in veterinary, public and environmental health sectors in your country

	1	2	3	4	5	
poor	◯	◯	◯	◯	◯	excellent

◯ No answer/I don't know

QUESTION 5.2

Are there recent (formal) initiatives to establish/strengthen intersectoral collaboration (at administrative/academic level) aimed to global advocacy of OH approach?

◯ Yes

◯ No

◯ No answer/I don't know

If yes, please briefly describe

Your answer

QUESTION 5.3

Which are the top three environmental, animal and human health issues in your country over the past 5 years?

Your answer

QUESTION 5.4

Please list a minimum of 3 institutions which coordinate/are responsible for OH activities in your country

Your answer

○ No answer/I don't know

QUESTION 5.5

In your opinion where and what are the gaps in One Health schemes?

Your answer

○ No answer/I don't know

QUESTION 5.6

In your opinion what is the level of awareness/perception of OH amongst citizens/consumers of your country?

	1	2	3	4	5	
None	○	○	○	○	○	Excellent

Whatinitiatives could be undertaken to develop/improve such awareness?

Your answer

6. End of questionnaire

Comments, other remarks if you would like to add

Your answer

Suggestions for improving the questionnaire

Your answer

Thank You for Completing Our Survey

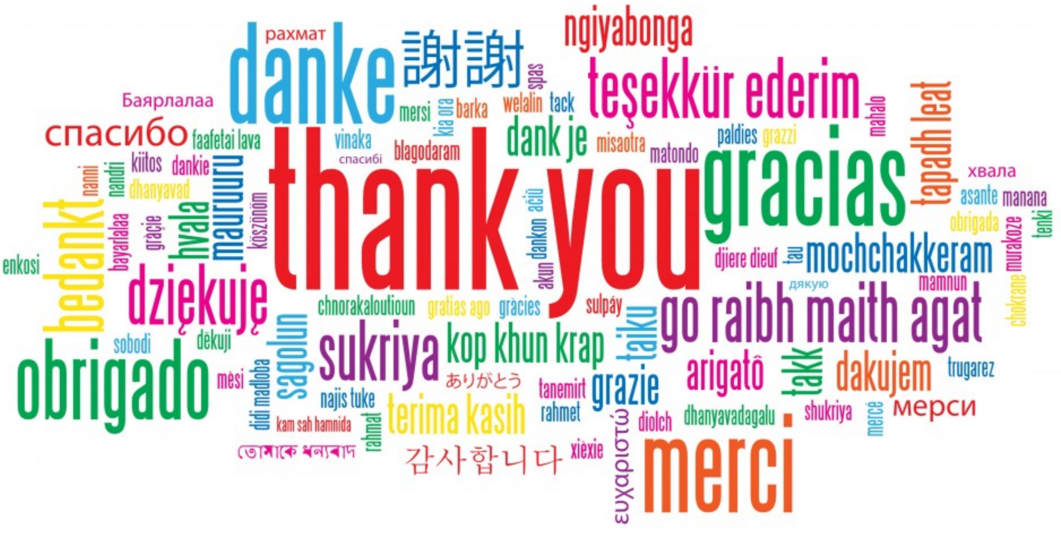

Network for Evaluation of One Health

cost
EUROPEAN COOPERATION IN SCIENCE AND TECHNOLOGY

EUR NEG VEC
European Network for Neglected Vectors and Vector-Borne Infections
